# Personality and Psychological Factors of Problematic Internet Gamers Seeking Hospital Treatment

**DOI:** 10.3389/fpsyt.2019.00583

**Published:** 2019-08-28

**Authors:** Wonshik Seong, Ji Sun Hong, Soyoung Kim, Sun Mi Kim, Doug Hyun Han

**Affiliations:** Department of Psychiatry, Chung-Ang University Hospital, Seoul, South Korea

**Keywords:** healthy internet use, internet gaming disorder, temperament and character, depression, attention deficit

## Abstract

**Introduction:** Previous studies on internet gaming disorder (IGD) have reported an association between personality traits and impulsive or problematic use of the internet or internet games, but the results obtained were inconsistent. Our study’s hypothesis was that personality traits are associated with the individual’s choice to play internet games, and psychological status of the individual is associated with seeking treatment for addictive behavior at a hospital.

**Method:** In the current study, individuals who reported excessive internet gaming and visited the hospital for treatment were enrolled and defined as the problematic internet gaming group; through advertisement, additional 138 individuals who were frequent gamers and 139 who were infrequent gamers were recruited. In a multiple logistic regression analysis of all participants’ data, a discrete set of hierarchical variables, with gaming preference (frequent gamers + problematic gamers) or problematic internet gaming as the dependent variable, was added to the demographic factors for model 1, personality traits for model 2, and psychological state for model 3.

**Results:** Temperament was a potential factor associated with internet gaming preference. Additionally, model 2, which comprised both demographic factors and personality traits, was a significant factor to enhance the predictability of internet gaming preference with maximum accuracy of 96.7%. Of the three models in the current study, model 2 and model 3 with combined model 2 and patient’s psychological status were associated with problematic internet gaming.

**Discussion:** The current study indicated that personality traits were potential factors associated with the individual’s preference for gaming. In addition, abnormal psychological status, especially, depressive mood and attention deficit, may lead individuals with problematic internet gaming to seek treatment at the hospital.

## Introduction

In 2017, 70.3% of Korean adolescents and adults, between 10 and 65 years of age, played internet games (1). Most studies on internet gaming have focused on the negative impact of games (2). However, two pioneering studies have suggested that playing video games may provide a positive influence (3). Green and Bavelier (4) reported that playing action video games markedly improved visual selective attention (4), and Sun et al. (3) reported that computer games enabled visuospatial abilities. In addition, several studies have suggested that video gaming has benefits related to cognitive (5), motivational (6), emotional (7), and social (8) behaviors. Due to the controversy regarding the advantages and disadvantages of video gaming, investigators are unsure of the classification of problematic internet gaming as a mental health disorder: King et al. (9) proposed the classification of internet gaming disorder (IGD) as a mental health problem (9), whereas Aarseth et al. (10) refuted such classification based on low disease criteria and negative stigma in moderate gamers (10).

Our aim was to determine human factors of the individual’s preference for online games, and causal factors of problematic gaming and those contributing to the decision to seek treatment at a hospital of individuals with excessive gaming.

Several previous studies have suggested that the game genre, and gamer’s personality traits, and psychological status may induce problematic internet gaming. However, there are few integrated studies on internet gaming, and hence, the factors of internet gaming preference, the gamer’s genre preferences, and etiology of IGD remain unclear (11). Laier et al. (12) reported that maladaptive personality traits were associated with symptoms of negative affectivity, detachment, and psychoticism in patients with IGD (12). Palaus et al. (13) reviewed the neural and cognitive features of patients with IGD and recommended additional research to assess the gamer’s characteristics and genre preferences to clarify etiology of the disorder (13).

Cloninger et al. (14) developed the temperament and character inventory (TCI); the psychobiological model suggested that both genetic and environmental factors influence an individual’s personality which is reflected in temperament and manifestation of character traits (14). TCI has been used for investigation of the neurobiological foundation of personalities (15), relationship with other personality models (16), and psychological well-being (17). Several studies focused on IGD have indicated an association between personality traits, impulsivity, and problematic internet game playing (18, 19). Kim et al. (18) reported that the severity of IGD was highest in role-playing games (RPG) than other game genres (18). In addition, the genres of RPG and real-time strategy were associated with high novelty-seeking (NS) and self-directedness (SD) scores. Cho et al. (19) reported that IGD participants had high SD and cooperativeness (Co) scores as well as low NS and self-transcendence (ST) scores (19). Based on known correlations between personality traits and internet gaming, studies with comparison between infrequent gamers, frequent gamers, and problematic gamers may reveal the effects of personality traits on internet gaming.

Previous reports have indicated that the psychological status, including comorbid conditions, lead to or stem from problematic internet game playing (20, 21, 22). A study including 263 patients who visited a hospital for IGD treatment reported that psychological factors, such as attention deficit and depressed mood, were the strongest predictors of IGD development (23). In addition, psychological status was a significant prognostic factor of IGD (20, 23). Han et al. (24) reported that attention-deficit hyperactivity disorder (ADHD) treatments showed side-effect of an increase in severity of IGD in children with concomitant ADHD and IGD (24, 25); in concordance, treatment of major depressive disorder (MDD) had effectiveness to improve both the impulsivity and IGD symptoms in patients with combined MDD and IGD (24). Based on the findings of previous studies, we considered that psychological problems may be an influencing factor for the decision to seek treatment in the hospital in individuals with excessive internet gaming.

Affinity for internet game and problematic internet gaming may be determined by individual factors, including characteristics and psychological status. In this study, we hypothesized that personality traits are associated with playing of internet games, and the psychological status is associated with seeking of treatment at a hospital in problematic internet gamers.

## Materials and Methods

### Participant Recruitment

The problematic gamer group was recruited from patients at an online-game-treatment clinic, while the healthy gamer group (frequent and infrequent gamers) was recruited from the general population through advertisement.

Of the 183 adult patients who visited the OO University Hospital Online Game Clinic and Research Center between January, 2015, and February, 2018, 167 were enrolled in the study as the problematic gamer group, and consent to participate in the study was obtained from all the subjects. Of the 167 patients enrolled, 152 (91.0%) successfully completed the research protocol. Inclusion criteria for the problematic gamer group were as follows: 1) at least 18 years of age; 2) fulfillment of IGD diagnostic criteria per Diagnostic and Statistical Manual of Mental Disorders, fifth edition (DSM-5) (26); and 3) young internet addiction scale score (YIAS) of >50.

Through advertisement for healthy game users or non-gamers at OO University and OO University Hospital, 302 adults were screened; among these, 21 adults had YIAS >50, and four adults played games >4 h/week and were excluded from the study. A total of 277 healthy game users were recruited with the following inclusion criteria: 1) at least 18 years of age, 2) without history of visit to hospital or counseling centers for resolution of problematic internet game play, 3) not satisfying the diagnostic criteria for IGD, and 4) YIAS of <50, and playing internet games ≤4 h/week. Based on the criteria of frequency of gaming and play time, 277 healthy game users were classified into two groups: frequent gamers (twice or more a week; >2 h/week but <4 h/week of online gaming) and infrequent gamers (once or more a week; ≤2 h/week of online gaming).

Finally, data was collected from 152 problematic gamers, 138 frequent gamers, and 139 infrequent gamers. Written informed consent forms were signed by all participants, and the study was approved by the Chung Ang University Hospital Ethics Committee IRB. All participants in both the gamer and infrequent-gamer groups were compensated with $20 USD on survey completion.

### Assessment Scales

Demographic data including the age, sex, years of education, and preferred genre of games were collected from all participants for subsequent evaluation. With regard to the genre of games, the subjects were asked to identify the genre of games they played from among the following: first-person shooter (FPS; e.g., rainbow series, Counter-Strike, and Overwatch), RPG (e.g., Final Fantasy series, World of Warcraft, and Diablo series), real-time strategy (RTS; e.g., Starcraft series, Warcraft series, and Company of Heroes series), and others.

YIAS was used to assess the severity of IGD symptoms (27), which is a self-reported questionnaire consisting of 20 questionnaire items scored on a Likert scale between 1 (rarely) and 5 (always) (28); the Cronbach’s α of YIAS was 0.89 (28).

TCI-revised-short version (TCI-RS) developed by Cloninger et al. (14) was used to assess temperament and personality (14). Specifically, the 140-item TCI-RS was developed to measure four temperamental factors including novelty seeking (NS), harm avoidance (HA), reward dependence (RD), and persistence (Pe), and three-character traits including self-directedness (SD), cooperativeness (Co), and self-transcendence (ST); the Cronbach’s α for the TCI-RS facet scales ranged from 0.70 to 0.88 (14). The T-scores of TCI-RS were applied in the analysis.

Both Beck Depressive Inventory (BDI)-II (29) and Beck Anxiety Inventory (BAI) (30) were used to evaluate mood and anxiety, respectively; the Cronbach’s α of the total scores of the BDI-II full version was 0.89 (31), and the internal consistency of BAI was 0.93 (30). Finally, the attention problem was assessed with the Korean version of DuPauls’ ADHDA Rating Scale (K-ADHD-RS) (32, 33) validated by So et al. (34) with an internal consistency ranging between 0.77 and 0.79 (34).

### Statistical analyses

The demographic characteristics, personality traits, and psychological states were analyzed using one-way analysis of variance (ANOVA) and chi-square tests. In a multiple logistic regression analysis involving all participants’ data, a discrete set of hierarchical variables, with gaming preference (frequent gamer group + problematic gamer group) as the dependent variable, was added to the demographic factors for model 1, personality traits for model 2, and psychological state for model 3; in addition, a discrete set of hierarchical variables, with problematic internet game play as the dependent variable, was added in the same three models. Finally, multiple logistic regression analysis with temperament and characteristics as the independent variables, and game genres as the dependent variables, was conducted to assess the correlation between personality traits and game genre.

### Ethics Statement

The research protocol was approved by the Institutional Review Board of Chung Ang University Hospital. The study was conducted in accordance with the ethical standards of the Helsinki Declaration of 1964 and subsequent amendments or similar ethical standards. Written informed consent was obtained from all participants.

## Results

### The Comparison in Demographic, Personality Traits, and Psychological Data Between the Three Groups

No significant differences in age, sex distribution, and years of education were observed between the infrequent-gamer, frequent-gamer, and problematic gamer groups. Despite absence of significant difference in game genre between the frequent-gamer and problematic gamer groups, the YIAS was higher in the problematic gamer group *versus* the frequent gamer group (**Table 1**).

**Table 1:** Comparison of the demographic, personality, and psychological data among the three groups.

	Infrequent gamer (IG) (n = 139)	Frequent gamer (FG) (n = 138)	Problem gamer (PG) (n = 152)	Statistics
Demographics				
Age	25.7 ± 5.1	25.8 ± 5.9	26.3 ± 5.5	F = .630; p = .53
Sex (male/female)	124/15	122/16	138/14	χ^2 =^ 0.46; p = 0.65
Years of education	12.6 ± 1.6	13.0 ± 1.8	12.8 ± 1.7	F = 2.32; p = .10
Genre (n, %)	–			
RPG	–	29 (21.2)	46 (30.1)	χ^2 =^ 3.09; p = 0.22
RTS	–	67 (48.2)	64 (42.3)	
FPS	–	19 (13.9)	20 (12.9)	
Others	–	23 (16.8)	22 (14.7)	
YIAS	–	36.5 ± 9.1	63.6 ± 10.4	F = 631.88; p < .01
Temperament and character inventory				
NS*	37.8 ± 8.6	52.2 ± 11.2	57.4 ± 10.8	F = 39.94; p < .01
HA*	36.5 ± 12.7	47.1 ± 11.5	55.9 ± 13.7	F = 76.18; p < .01
RD*	37.7 ± 9.8	44.8 ± 11.1	43.3 ± 11.5	F = 13.14; p < .01
Pe*	37.9 ± 11.6	43.9 ± 11.2	40.1 ± 10.2	F = 9.93; p < .01
SD*	45.5 ± 12.7	40.8 ± 11.8	41.1 ± 12.0	F = 18.17; p < .01
Co*	50.2 ± 12.9	47.6 ± 11.3	44.7 ± 9.9	F = 8.11; p < .01
ST*	34.9 ± 8.7	41.5 ± 9.8	43.5 ± 9.1	F = 141.54; p < .01
Psychological status				
BDI*	5.9 ± 5.0	5.5 ± 3.6	13.4 ± 6.4	F = 101.90; p < .01
BAI*	3.9 ± 4.5	5.6 ± 5.0	12.1 ± 10.6	F = 39.97; p < .01
K-ADHD-RS*	4.3 ± 2.9	5.4 ± 3.7	19.4 ± 10.8	F = 46.52; p < .01

*Statistically significant; NS, novelty seeking; HA, harm avoidance; RD, reward dependence; Pe, persistence; SD, self-directedness; Co, cooperativeness; ST, self-transcendence; BDI, Beck Depressive Inventory; BAI, Beck Anxiety Inventory; K-ADHD-RS, Korean version of the Dupauls’ ADHDA Rating Scale; Post hoc, NS: IG < FG < PG; HA: IG < FG < PG, RD: IG < FG = PG; Pe: IG = PG < FG; SD: IG > FG = PG; Co: IG > PG; ST: IG < FG = PG; BDI: IG = FG < PG; BAI: IG = FG < PG; K-ADHD-RS: IG = FG < PG.

Significant differences in the NS, HA, RD, Pe, SD, Co, and ST scores between the three groups were revealed. Specifically, the NS and HA scores were ordered as follows: problematic gamer group > frequent-gamer group > infrequent-gamer group; the RD and ST scores were higher in both the frequent-gamer and problematic gamer groups than in the infrequent-gamer group; the SD scores were lower in both the frequent-gamer and problematic gamer groups than in the infrequent-gamer group; whereas, the Co scores were higher in the infrequent-gamer group than in the problematic gamer group (**Table 1**).

Finally, significant differences were observed in the BDI, BAI, and K-ADHD-RS scores between the three groups, and highest values were obtained in the problematic gamer group (**Table 1**).

### Hierarchical Logistic Regression Analysis for Gaming Preference

Of the three models in the current study, model 2 and model 3 were significantly associated with gaming preference. Based on achievement of highest step chi-square value and improvement in classification accuracy, temperament and character were considered as the strongest influencing factors of gaming preference. Additionally, model 2, which comprised both model 1 and personality traits, significantly enhanced the predictability of gaming preference, reaching an accuracy of 96.7%. In contrast, model 3, which included model 2 and the psychological status, significantly increased the predictability of gaming preference, showing a 98.4% prediction accuracy. According to the Wald statistics for all independent variables, higher NS, HA, and RD were significant predictors of gaming preference (**Table 2**).

**Table 2:** Effects of both personality and psychological factors on internet gaming.

Independent variables		Model 1			Model 2			Model 3		
		B	Wald	OR	B	Wald	OR	B	Wald	OR
Demographic factors	Age	.016	.491	1.026	−.089	1.468	.915	−.106	1.737	.889
	Sex	−.428	.527	.652	−.545	.040	.580	−2.085	.086	.124
	Years of education	.189	6.104	1.209^*^	−.032	.013	.968	.012	.001	1.012
TCI	NS				.391	19.588	1.478**	.408	17.421	1.504**
	HA				.077	11.748	1.472**	.408	10.241	1.503**
	RD				−.013	2.373	1.081	.134	4.812	1.144*
	PE				.438	.077	.987	−.016	.103	.985
	SD				−.050	2.266	0.952	−.057	2.862	0.944
	CO				.084	2.531	.915	−.126	3.657	.882
	ST					2.696	1.087	.106	2.856	1.112
Psychological status	BDI							.196	2.681	1.216
	BAI							.144	1.953	1.155
	K-ADHD-RS							.026	.275	1.026
Indices	Model 0	Model 1			Model 2			Model 3		
−2LL	490.30	419.29			48.55			41.81		
Model χ^2^/p	N/A	8.0/0.06			378.76/ < 0.01			385.49/ < 0.01		
Nag 2	N/A	0.031			0.936			0.945		
Class accur	73.0	73.0			96.7			98.4		

**p < 0.01; *p < 0.05; −2LL, −2 log likelihood; Nag R2, Nagelkerke’s R2; class accur, classification accuracy; dependent factor: gaming preference (frequent gamer group + problematic gamer group); novelty seeking, NS; harm avoidance, HA; reward dependence, RD; persistence, Pe; self-directedness, SD; cooperativeness, Co; self-transcendence, ST; Beck Depressive Inventory, BDI; Beck Anxiety Inventory; BAI; Korean version of the Dupauls’ ADHDA Rating Scale, K-ADHD-RS.

### Effects of Temperament and Character on the Choice of Internet Game Genre in Individuals’ Internet Gaming

High NS scores predicted either the RPG or the RTS genre in the frequent-gamer group, whereas low HA scores predicted the FPS genre in the same group. There were no factors of temperament or character that significantly predicted a special game genre in the problematic game group (**Table 3**).

**Table 3:** Effects of personality traits on the choice of internet game genre in individuals with internet game-play behavior.

	Role-playing game				Real-time strategy game				First-person shooting game				Others			
	B	Wald	P	Exp(B)	B	Wald	P	Exp(B)	B	Wald	P	Exp(B)	B	Wald	P	Exp(B)
NS	.055	4.759	.029	1.056*	.058	4.152	.042	1.059^*^	−.018	.385	.535	.982	−.022	1.319	.251	.978
HA	.015	.304	.581	1.025	.012	.171	.679	1.012	−.058	3.478	.041	.943^*^	.014	.437	.509	1.014
RD	−.002	.011	.917	.998	−.014	.268	.604	.986	−.057	3.822	.062	.945	.003	.028	.866	1.003
PE	−.008	.087	.767	.992	−.025	.798	.372	.975	−.022	.549	.459	.978	.032	2.454	.117	1.033
SD	.019	.431	.511	1.019	.049	2.255	.133	1.051	−.045	1.449	.229	.956	−.018	.587	.444	.982
CO	.024	1.087	.297	1.024	.020	.553	.457	1.020	−.011	.106	.745	.989	−.021	1.085	.298	.979
ST	.020	.549	.459	1.020	−.024	.823	.364	.977	.039	1.625	.202	1.040	−.015	.568	.451	.985

*p < 0.05; dependent factor: Role playing game, Real strategy game, First person shooting game, other game genres, NS, novelty seeking; HA, harm avoidance; RD, reward dependence; Pe, persistence; SD, self-directedness; Co, cooperativeness; ST, self-transcendence

### Hierarchical Logistic Regression Analysis for Problematic Internet Gaming

Of the three models employed in the current study, model 2 and model 3 were significantly associated with problematic internet gaming. With the highest step chi-square value and improvement in classification accuracy, the psychological status was the main factor related to problematic internet gaming. Individuals with excessive internet game usage who visited hospital for treatment in the current study were defined as the problematic internet gaming group. Model 2, which included model 1 and personality traits, was a significant factor to increase the predictability of problematic internet gaming with an accuracy of 66.4%. In contrast, model 3, which comprised both model 2 and psychological status, was a significant factor to increase the predictability of problematic internet gaming, with a prediction accuracy of 83.4%. Based on Wald statistics for all independent variables, higher BDI and K-ADHD-RS scores were significant predictors of problematic internet gaming (**Table 4**).

**Table 4:** Effects of both personality and psychological factors on problematic internet gaming.

Independent variables		Model 1			Model 2			Model 3		
		B	Wald	OR	B	Wald	OR	B	Wald	OR
Demographic factors	Age	.031	1.613	1.032	.034	1.547	1.035	.019	.270	1.019
	Sex	−1.144	1.767	.318	−1.540	2.531	.214	−1.520	1.075	.219
	Years of education	−.021	.077	.979	−.028	.110	.972	.053	.223	1.054
	Genre	−.231	2.961	.794	−.223	2.158	.800	−.156	.576	.856
TCI	NS				.011	.415	1.011	.018	.613	1.018
	HA				.030	3.145	1.030	−.007	.090	.993
	RD				.000	.000	1.000	−.002	.012	.998
	PE				.010	.312	1.010	.028	1.415	1.029
	SD				−.036	1.163	.945	−.033	1.580	.967
	CO				.004	.057	1.004	.010	.183	1.010
	ST				.013	.513	1.013	−.016	.389	.984
Psychological status	BDI							.331	5.582	1.393**
	BAI							.004	.019	1.004
	K−ADHD−RS							.045	5.530	1.046*
Indices	Model 0	Model 1			Model 2			Model 3		
−2LL	334.42	327.52			280.26			178.35		
Model χ^2^/p	N/A	6.38/.17			53.64/ < .01			155.54/< .01		
Nag 2	N/A	.035			.266			.634		
Class accur	53.5	53.5			66.4			83.4		

**p < 0.01; *p < 0.05; −2LL, −2 log likelihood, Nag R2, Nagelkerke’s R2; class accur, classification accuracy; dependent factor: problematic internet game playing, NS, novelty seeking; HA, harm avoidance; RD, reward dependence; Pe, persistence; SD, self-directedness; Co, cooperativeness; ST, self-transcendence; BDI, Beck Depressive Inventory; BAI, Beck Anxiety Inventory; K-ADHD-RS, Korean version of the Dupauls’ ADHDA Rating Scale.

## Discussion

### Personality Traits of Internet Gamers

ANOVA analysis revealed significant differences of all sub-items of the TCI among the three groups. The hierarchical logistic regression analysis for internet gaming revealed that temperament and character, including higher NS, HA, and RD, were the strongest influencing factors of internet gaming; whereas, both the NS and RD scores were positively associated with extroversion and openness (16), and the HA score was inversely associated with extroversion (16). Our results of personality traits except HA were similar to those of the study of Teng, (35) to compare internet gamers to non-internet gamers; that author reported that gamers obtained higher scores for openness, conscientiousness, and extroversion than non-gamers. In contrast to low HA obtained in that study, in our study, the gamers obtained high NS and HA. Lee et al. (36) reported that IGD patients without improvement showed higher HA scores, compared to IGD patients with improvement. The collective data of the previous and present studies suggest that individuals who enjoy internet gaming present the personality traits of openness and happiness; however, there is conflicting data with regard to extroversion and conscientiousness that are controversial.

### Effects of Temperament and Character on the Choice of Internet Game Genre Among Internet Gamers

The results of multiple logistic analysis on the effect of TCI on genre preference in the healthy gamer group (frequent gamer + infrequent gamer) revealed that the NS scores were positively associated with RPG and RTS genre preferences, and the HA scores were negatively correlated with FPS genre preference, which is in agreement with the result of higher NS scores in RPG and FPS gamers of our previous study including 565 adolescent internet gamers (18). Many games of those genres may require relationship-building to promote social online setting for game play, specially that RPG and FPS games require social interaction for group play, which may explain the positive correlation between extroversion and games of those genres.

A study has indicated that NS scores were related to cravings and impulsive behaviors in individuals with chemical addictions (37), and several studies have suggested that high NS scores attained for RPG games was associated with higher internet addiction scores of the subjects as compared to those for other game genres (19). However, Peever et al. (38) reported opposite result of negative correlation between RPG and RTS games and extroverted personality in their study on personality and video game genre preference in 566 participants (38), and another study reported that low SD scores were related to gamers playing FPS games (39). The difference in results among the studies may be explained by variations in the designs of the games even of the same genre, age distribution (adults and adolescents *vs*. adults only), and assessment tool used for personality traits.

### Psychological Status of Problematic Internet Gamers

ANOVA analysis of data among the three groups revealed that the BDI, BAI, and K-ADHD-RS scores were highest in the problematic gamer group. Moreover, in the hierarchical logistic regression analysis for problematic internet gaming, the psychological status of gamers strongly predicted problematic internet gaming. Both mood statuses under BDI and attention deficit according to K-ADHD-RS scores were positively correlated to problematic internet gaming. Several previous studies have reported that problematic internet gaming may be associated with either MDD or ADHD (40, 41, 42). Ha et al. (40) reported that a large number of adolescents with IGD had comorbidities of both ADHD and MDD (40). Carli et al. (41) conducted a literature review related to investigations of comorbidities of pathological internet use and reported that 75 and 100% of the studies had findings of significant correlation with MDD and ADHD symptoms, respectively (41). Moreover, Han et al. (42) conducted a study of the brain in 78 adolescents with IGD and suggested that the over connectivity between the default and executive control networks may be due to psychiatric comorbidities, including MDD and ADHD, and these comorbidities were key predictors of the IGD prognosis at long term follow-up (43). Finally, lower BDI and K-ADHD-RS scores at baseline were associated with better recovery from the IGD, whereas higher BDI and K-ADHD-RS scores at baseline required extended treatment (43). Therefore, in individuals who are hospitalized for treatment for problematic internet gaming, the clinicians should consider psychiatric conditions including ADHD or MDD.

### Limitations

Our study has some limitations. Although we investigated the relationships between personality, and psychological status, and problematic internet gaming, we did not consider other factors, including social support and family cohesion. Several reports suggested that parental rejection may cause IGD (44), and cognitive emotion is a potential regulatory factor of vulnerability for IGD (45). Moreover, simplifying the game genres within four categories may affect the results of the analysis. Second, the problematic gamers in the current study had already been hospitalized for treatment and comprised only a small portion of the problematic gamers in society; hence, generalizing of the results requires caution. Finally, our study included only few female participants. Future studies including family and social environmental factors, larger number of women, and additional game genres are needed.

### Conclusion

The current study has a strength of analysis of clinical samples. In the comparisons among the three groups of infrequent gamers, frequent gamers, and problematic gamers, both personality and psychological statuses had significant effects on internet gaming preference and pathological gaming. Overall, the personality traits of temperament and character were associated with gaming preference, and problematic internet gaming was more strongly correlated with the psychological status, specifically depressive mood and attention deficit.

## Ethics Statement

All participants signed a written informed consent form and the study was approved by the Chung Ang University Hospital Ethics Committee IRB.

## Author Contributions

JH, SYK, and DH contributed to patient recruitment, and data collection and processing. WS, SMK, and DH analyzed the data. All authors participated in writing of the manuscript, and contributed to the intellectual aspect. All authors read and approved the final manuscript.

## Funding

This study was supported by a grant from the Korean Creative Content Agency (KOCCA17-80).

## Conflict of Interest Statement

The authors declare that the research was conducted in the absence of any commercial or financial relationships that could be construed as a potential conflict of interest.
